# Percutaneous puncture of renal calyxes guided by a novel device coupled with ultrasound

**DOI:** 10.1590/S1677-5538.IBJU.2014.0586

**Published:** 2015

**Authors:** Chen Jen Chan, Victor Srougi, Fabio Yoshiaki Tanno, Ricardo Duarte Jordão, Miguel Srougi

**Affiliations:** 1 Seção de Endorologia e Videolaparoscopia, Divisão de Urologia, Hospital das Clínicas, Universidade de São Paulo, Escola de Medicina, São Paulo, Brasil

**Keywords:** Punctiometer, percutaneous access, kidney punction, nephrolitotripsy, ultrassound

## Abstract

**Purpose:**

To evaluate the efficiency of a novel device coupled with ultrassound for renal percutaneous puncture.

**Materials and Methods:**

After establishing hydronephrosis, ten pigs had three calyxes of each kidney punctured by the same urology resident, with and without the new device (“Punctiometer”). Time for procedure completion, number of attempts to reach the calyx, puncture precision and puncture complications were recorded in both groups and compared.

**Results:**

Puncture success on the first attempt was achieved in 25 punctures (83%) with the Punctiometer and in 13 punctures (43%) without the Punctiometer (p=0.011). The mean time required to perform three punctures in each kidney was 14.5 minutes with the Punctiometer and 22.4 minutes without the Punctiometer (p=0.025). The only complications noted were renal hematomas. In the Punctiometer group, all kidneys had small hematomas. In the no Punctiometer group 80% had small hematomas, 10% had a medium hematoma and 10% had a big hematoma. There was no difference in complications between both groups.

**Conclusions:**

The Punctiometer is an effective device to increase the likelihood of an accurate renal calyx puncture during PCNL, with a shorter time required to perform the procedure.

## INTRODUCTION

Minimally invasive surgical techniques have gained increasing prominence because of their advantages of shortening the convalescence period and producing less pain during postoperative recovery. In accordance with this trend, percutaneous techniques have been developed to treat urinary tract stones that previously required open surgery with long and painful incisions.

During percutaneous nephrolithotomy (PCNL) (1–3), the urinary tract is accessed by a renal puncture, followed by dilation and the use of optical instruments to find and remove the stones. Percutaneous access to the excretory pathway is an essential step, but it is not always easily achieved, and more than one attempt is frequently necessary. Furthermore, the procedure is often performed using fluoroscopic guidance, which exposes the surgeon and patient to radiation (4). The procedure can also be performed using ultrasound (5, 6), with the advantage of avoiding radiation but the disadvantage of requiring the presence of an ultrasound expert.

To facilitate renal calyx puncture, we developed an apparatus called the “Punctiometer” for use with ultrasound guidance. This device can be extremely helpful, especially in the presence of ureteral obstruction or when it is not possible to position a ureteral catheter to inject the excretory pathway with contrast before conventional fluoroscopic puncture. The purpose of the current study is to evaluate the efficacy of the Punctiometer device in aiding percutaneous puncture of renal calyxes in a pig model.

## MATERIALS AND METHODS

This experimental study was approved by the Ethics Committee in Research of the University of São Paulo School of Medicine (Protocol 432/11). It was performed in the CEPEC-Center of Training and Research in Surgery “Vicky Safra” of Faculty of Medicine University of São Paulo (FMUSP) from December 2012 to January 2013. Ten 30-kg Duroque pigs of the MC60 lineage were used. The animals were anesthetized with 100mg ketamine, 400mg xylazine, 15mg midazolam, 250mg thiopental, 4mg pancuronium, and 0.05mg fentanyl; intubated and mechanically ventilated with 100% O2; and placed in the lateral position.

All kidneys were punctured using ultrasound guidance, after blinded randomization to either the Punctiometer or no Punctiometer Groups. Laparoscopic access was used to ligate the ureters bilaterally to promote mild dilation of the excretory pathways. The ligation was executed with a cotton thread and endocorporeal knot in the proximal portion of the ureter, ensuring total luminal occlusion. Approximately 20 minutes after, the animals were then positioned in the prone position for the renal punctures, which were performed by a senior urology resident with previous training in percutaneous surgery. A 2-0 nylon thread was used as a guidewire to identify the exact puncture location in the calyx. Three calyxes of each kidney were punctured (superior, middle, and inferior). Bilateral nephrectomy was subsequently performed to verify the position of the guidewire. If the nylon guidewire was lost, the puncture was excluded from the final evaluation. At the end of the procedure, the pigs were sacrificed with 20mL potassium chloride and discarded in accordance with the local law (“RSS-Resíduos Sólidos de Serviços de Saúde (Solid residuals of Health Services)”-São Paulo (7).

The final evaluation involved determination of the following: number of attempts to reach each calyx, precision index score, total time to perform the procedure for each kidney, and occurrence of complications. The precision index score was rated on a 0 to 2 point scale: 2 points, reached the collecting system in the target calyx; 1 point, reached the renal pelvis; or 0 points, did not reach the collecting system. Complications were rated on a 1 to 4-point scale: 1 point, small hematoma; 2 points, moderate hematoma; 3 points, big hematoma; or 4 points, huge hematoma. The definition of the hematoma grade was subjective and evaluated by the surgeon who performed the puncture.

### Punctiometer

**Figure 1:**
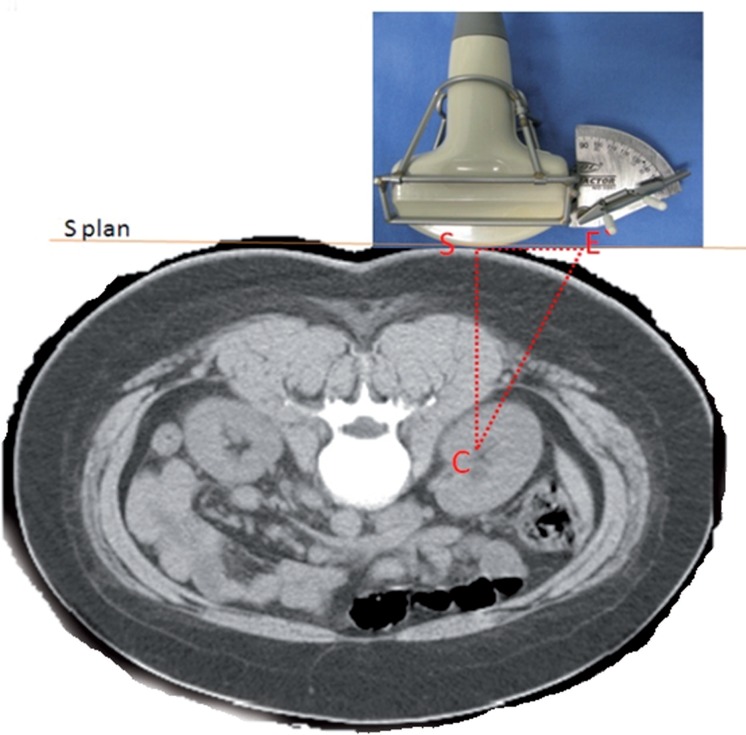
vertices of the initial right triangle: the target calyx (c); the “apex” of ultrasound transducer in contact with the skin, perpendicular to the aimed calyx (s); and the orthogonal projection of the center of the protractor in the skin plane (E').

**Figure 2:**
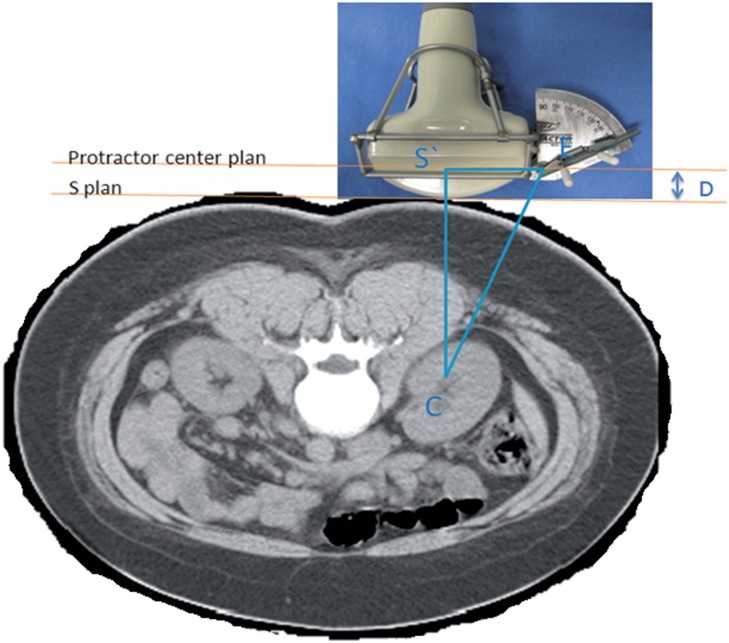
Formation of the right triangle. S'C: Distance from the target calyx (C) to its orthogonal projection on the plane of the center of the protractor (S'). S'E: Distance between point S' and the center of the protractor (E). CE: Distance from the target calyx (C) to the center of the protractor (E). D: Distance between the planes.

Use of the Punctiometer involves the general principle of first identifying a triangle (Figure-1) formed by the target renal calyx (C), the “apex” of the ultrasound transducer at a right angle with the calyx (S), and the orthogonal projection of the protractor with the skin (E'). Using a triangular Figure, it is possible to calculate the hypotenuse when it is known the length of the opposite and adjacent sides. From the triangle in Figure-1, we derive the triangle in Figure-2, which is the triangle actually used for the puncture. One of the sides of this second triangle is the distance between the center of the protractor (E) and the point of the orthogonal projection of the target calyx to the surface of the protractor (S'E). This distance is fixed in the apparatus. The other side of the triangle is the distance between the calyx and the skin where there is contact with the apex of the ultrasound transducer. This corresponds to the distance measured by ultrasound, added to the distance (D) from the ultrasound apex to the center of the protractor (S'C).

### Statistical analyses

Descriptive analyses of the quantitative data with a normal distribution included determination of the means, with their respective standard deviations (±SD). Qualitative data were presented as frequencies and percentages. We used Student's t-tests to compare quantitative variables. Qualitative variables were evaluated by likelihood ratio tests for comparing proportions. For all inferential analyses, we considered the probability of a type I error (α level) of 0.05 to be statistically significant. Statistical analyses were performed using SPSS software, version 21 (SPSS 21.0 for Windows) (8).

## RESULTS

After randomization, 30 calyxes were successfully punctured in each Group (10 pigs), with the number of attempts ranging from 1 to 5. The average diameter of the calyxes measured by ultrasound was 9.1mm (7-17mm) in the Punctiometer Group and 10.5mm (5-17mm) in the no Punctiometer Group.

Puncture success on the first attempt was achieved in 25 punctures (83%) with the Punctiometer and in 13 punctures (43%) without the Punctiometer (p=0.011). The guidewire was lost in 1 calyx in the Punctiometer Group and in 5 calyxes in the no Punctiometer Group; these punctures were not included in the final analyses. A precision index score of 2 was noted in 21 of the 29 remaining punctures (72%) in the Punctiometer Group and in 13 of the 25 remaining punctures (52%) in the no Punctiometer Group (p=0.028). The mean time required to perform three punctures in each kidney was 14.5 minutes with the Punctiometer and 22.4 minutes without the Punctiometer (p=0.025).

**Figure 3:**
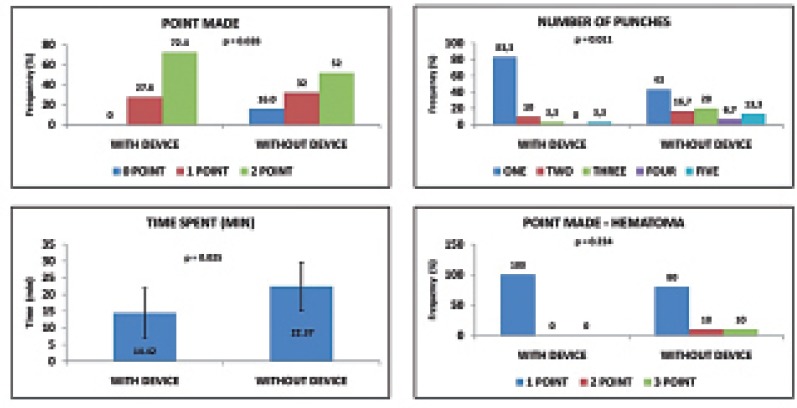
Comparison of frequencies and means between the Punctiometer and no Punctiometer groups: A) Precision index; B) number of puncture attempts required; C) mean time required to perform three punctures in each kidney; D) grade of complications (which were all hematomas).

There were no lesions produced in other organs. The only complications noted were renal hematomas of an intensity of 1 to 3 points. In the Punctiometer Group, all kidneys had small hematomas (designated as 1 point). In the no Punctiometer Group, 8 kidneys (80%) had small hematomas (1 point), 1 (10%) had a medium hematoma (2 points), and 1 (10%) had a big hematoma (3 points). The percentage of 1-point hematomas did not differ significantly between the two groups (p=0.224). Results are summarized in Table-1 and Figure-3

**Table 1:** Results of the puncture procedures in the punctiometer and no punctiometer groups.

	Punctiometer		p-value
	With	Without	
Calyxes with a successful 1st puncture	25 (83%)	13 (43%)	0.011
Calyxes with a precision index rating of 2	21 (72%)	13 (52%)	0.028
Time for three punctures in each kidney (min)	14.42±7.39	22.37±7.21	0.025
Kidneys with 1-point complications	10 (100%)	8 (80%)	0.224

Data are mean±standard deviation or number (percentage)

## DISCUSSION

Percutaneous access to the excretory system is a relatively easy procedure when the system is clearly dilated. However, it may be challenging when there is minimal or no dilation, as is frequently seen in patients with renal stones. The use of ultrasound to guide renal puncture may be valuable in this situation. In 1974, Pedersen and colleagues (9), were the first to describe the use of ultrasound during percutaneous nephrostomy, obtaining a success rate of 75%. Other studies addressing access to the excretory system have been published, most of which have reported on the accuracy of the technique used (10) without mentioning the number of attempts necessary for a successful puncture. Agostini et al. (11) reported that 9.2% of patients required more than one puncture attempt, and Krombach et al. (12) noted that use of a magnetic field-based navigation device to guide the puncture in a porcine model failed in 2 of 12 kidneys.

**Figure 4:**
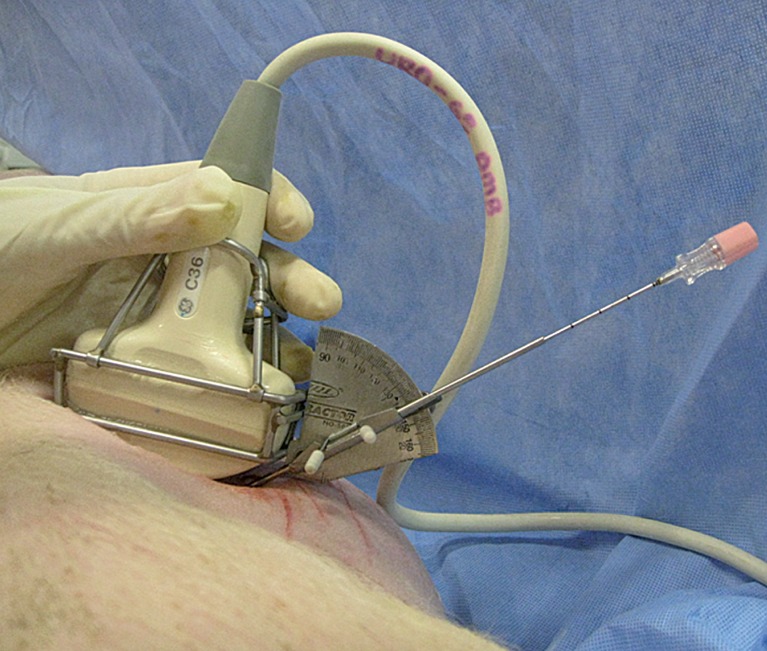
Ilustration of the punctiometer in use.

In our study, we successfully reached the excretory system in all cases, whether or not we used the Punctiometer (Figure-4). This high success rate may be due to the relatively short distance between the skin and renal calyxes in the pig, compared to humans. Most punctures were successful on the first attempt, but some required up to 5 attempts, which was inferior to the success rate reported by Agostini et al. (11). The discrepancy may be explained by the presence of urinary tract obstruction in the Agostini series, whereas in our study, the mean diameter of the calyxes was 9 to 10mm. Our findings regarding the need for more than one puncture attempt with the Punctiometer are comparable to those reported by Krombach and colleagues (12) using the ultrasound and magnetic field-based navigation device.

Regarding the precision index, we could find no previously published similar study with which to compare our results. Punctures were more precise in the Punctiometer Group than in the no Punctiometer Group. The difference was statistically significant, thereby suggesting that the device was effective in improving the likelihood of success of the puncture procedure.

Previously, Agostini et al. (11) reported that the time to place a percutaneous nephrostomy catheter was 7 to 15 minutes. Karim and colleagues (6) reported a longer time, 39 (25-55) minutes, to perform the same procedure. In our study, the time required to perform three punctures in each kidney was 14.4 minutes with the Punctiometer and 22.4 minutes without the Punctiometer, representing mean times of 4.8 minutes and 7.5 minutes per calyx in both Groups, respectively. Use of the Punctiometer was associated with significantly shorter renal puncture times. These results may be attributed to our use of only one guidewire in each calyx, without placing a nephrostomy catheter.

The most common complications of renal puncture accessed percutaneously are unintentional perforation of abdominal organs or pleura, and bleeding from renal or the great vessels. Although the risk of complications is small (6, 13, 14), the risk is higher when there is minimal or no dilation of the excretory system. In the present series, we observed no organ or pleura perforation and no bleeding arising from the great vessels; our complications were limited to renal bleeding. The difference in rate of 1 point hematomas between the Punctiometer and no Punctiometer Groups was not significant and the overall complication score was low in both Groups. However, it is possible that the short time between the punctures and nephrectomy may have limited the size of the observed hematomas.

## CONCLUSIONS

The renal Punctiometer is an effective device to increase the likelihood of an accurate renal calyx puncture during PCNL, with improved precision and a shorter time required to perform the procedure.
